# Predicting the onset of myopia in children: results from the CLEERE study

**DOI:** 10.1186/s12886-021-02036-9

**Published:** 2021-07-14

**Authors:** Donald O. Mutti, Lisa A. Jordan, Karla Zadnik

**Affiliations:** The Ohio State University College of Optometry, 338 West 10th Avenue, Columbus, OH 43210-1280 USA

**Keywords:** Myopia, Epidemiology, Risk factors, Children

## Abstract

Research often attempts to identify risk factors associated with prevalent disease or that change the probability of developing disease. These factors may also help in predicting which individuals may go on to develop the condition of interest. However, risk factors may not always serve as the best predictive factors and not all predictive factors should be considered as risk factors. A child’s current refractive error, parental history of myopia, and the amount of time children spend outdoors are excellent examples. Parental myopia and time outdoors are meaningful risk factors because they alter the probability of developing myopia and point to important hereditary and environmental influences. A child’s current refractive error points to no particular mechanism and is therefore a poor risk factor. However, it serves as an excellent predictive factor for identifying children likely to develop future myopia. Risk factors may explain how a child reached a particular level of refractive error, but knowledge of that history may not be needed in order to make an accurate prediction about future refractive error. Current refractive error alone may be sufficient. This difference between risk factors and predictive factors is not always appreciated in the literature, including a recent publication in *BMC Ophthalmology*. This letter attempts to make that distinction and to explain why parental myopia and time outdoors are significant risk factors in the Collaborative Longitudinal Evaluation of Ethnicity and Refractive Error, yet are not significant for predicting future myopia in a multivariate model that contains current refractive error.

We wish to point out an error we believe was made by Grzybowski and co-workers in their January 2020 paper in *BMC Ophthalmology,* “A review on the epidemiology of myopia in school children worldwide” [1]. One of their citations was from our National Institutes of Health-funded, US-based Collaborative Longitudinal Evaluation of Ethnicity and Refractive Error (CLEERE) Study: “Prediction of Juvenile-Onset Myopia” by Zadnik et al. in the June 2015 issue of *JAMA Ophthalmology* [2]. The following statement made in the review is an inaccurate interpretation of that paper that we would like to correct: “Less hyperopic and more myopic refractive error at the ages of 7 to 13 years was consistently associated with myopia onset, while having myopic parents, near work and time outdoors were not.”

Table 2 in Zadnik et al. (2015) clearly shows significant odds ratios for increased risk of becoming myopic associated with having one or two myopic parents compared to having none at each of the elementary school grades 1, 3, and 6 (ages 6, 8, and 11, respectively). Significant protective odds ratios are also shown for time outdoors at each grade/age. As correctly stated in the review, more time spent in near work was not a significant risk factor for becoming myopic [2]. The point of this paper from CLEERE was to develop a **predictive** model, not a risk factor model per se, for myopia onset. Factors were evaluated for their **predictive** value compared to knowing a child’s current refractive error. Predictive models and risk factor models may sound like they fulfill similar objectives, but their purposes should be seen as distinct. Risk factor analyses examine data for associations with disease outcomes, prevalent disease in cross-sectional studies or risk of developing disease in longitudinal data. Associations may shed light on disease mechanisms and possible mitigation strategies. Time outdoors reducing the risk of myopia onset and programs increasing children’s outdoor activity are excellent current examples. The goal of predictive models is more specific, to identify the individual at risk for disease. Risk factors may not necessarily be predictive factors and not all predictive factors are meaningful risk factors. Current refractive error is not usually included in a model of risk factors because it is uninformative about mechanisms; it does, however, serve as an excellent predictor of future myopia. Parental myopia and time outdoors had significant effects on myopia risk in CLEERE, but they were not significant in the multivariate results because they did not add predictive information compared to refractive error itself. Risk factors may explain how refractive error came to be in a range at risk for myopia onset, but may not add predictive information independent from refractive error itself. Put another way, having myopic parents and spending less time outdoors increased the chances that children would have a refractive error close to the “myopia cliff” placing them at higher risk of onset, but, having already contributed to refractive error, provided no additional predictive information. Knowing the refractive error was all that was needed to make the future myopia prediction. Knowing the risk factors that led up to that less hyperopic refractive error was not necessary.

We feel it is important to emphasize this point, that not all risk factors are predictive factors, because this error in interpretation has been made before. Xiong et al. made the same error in their meta-analysis with the statement “Zadnik et al. [2] did not found [sic] an association between time outdoors and risk of myopia onset in multivariate models” [3]. A more accurate statement would have been that Zadnik et al. [2] found that more time spent outdoors reduced the risk of the onset of myopia across a wide range of childhood ages.

## Data Availability

Not applicable.

## Abbreviation

CLEERE: The Collaborative Longitudinal Evaluation of Ethnicity and Refractive Error
